# Application of the nephrotoxic serum nephritis model in glomerulonephritis research

**DOI:** 10.1186/s10020-026-01460-1

**Published:** 2026-04-02

**Authors:** Jiayu Song, Sanyan Xu, Weihua Liu, Jingyi Sheng, Yongfeng Zhao, Shuzhen Li, Zhanjun Jia, Wei Gong

**Affiliations:** 1 Department of Nephrology, Children’s Hospital of Nanjing Medical University, Nanjing, 210008 China; 2 Department of Pediatric Nephrology, The Second Affiliated Hospital of Nanjing Medical University, Nanjing, 210003 China; 3 Nanjing Key Laboratory of Pediatrics, Children’s Hospital of Nanjing Medical University, Nanjing, 210008 China; 4 Jiangsu Key Laboratory of Early Development and Chronic Diseases Prevention in Children, Nanjing Medical University, Nanjing, 210029 China

**Keywords:** Glomerulonephritis, Nephrotoxic serum, Antibody deposition, NTN mouse model, Glomerular injury

## Abstract

Glomerulonephritis represents a significant public health challenge and frequently progresses to end-stage renal disease, which requires dialysis and ultimately organ transplantation. Animal models are crucial tools in biomedical research, providing experimentally feasible approaches to investigate the cellular and molecular mechanisms underlying disease pathogenesis. This review focuses on the nephrotoxic serum nephritis (NTN, also referred to as nephrotoxic nephritis) model, a glomerulonephritis animal model induced by nephrotoxic serum, which recapitulates key pathological features of immune-mediated glomerular diseases. We provide an overview of the model’s historical development, induction methods, renal pathological characteristics, and applications in the study of glomerulonephritis, lupus nephritis, renal fibrosis, among others. The target audience of this review comprises investigators in nephrology and immunology who employ, or are considering the use of, experimental models to study antibody-mediated glomerular injury.

## Introduction

In the early twentieth century, Richard M. Pearce and his colleagues discovered that serum obtained from rabbits immunized with dog kidneys was capable of inducing proteinuria in other dogs. They therefore concluded that the serum was nephrotoxic and could induce nephritis (Pearce and Sawyer 1908). Subsequently, Joseph E. Smadel summarized the methods for inducing experimental nephritis using anti-kidney serum and its pathological features, which were considered comparable to those observed in human nephritis (J E S. 1936). At the end of the twentieth century, molecular techniques further identified specific antibodies, such as anti-glomerular basement membrane (GBM)-type IV collagen antibodies, within the nephrotoxic serum (Chugh et al. 2001). Owing to its ability to replicate key pathological features of human glomerulonephritis, the nephrotoxic serum-induced animal model, named nephrotoxic serum nephritis (NTN) has since become a fundamental tool in the study of kidney diseases, particularly glomerulonephritis. Glomerulonephritis encompasses a range of immune-mediated disorders with complex pathogenic mechanisms that remain incompletely understood (Chadban and Atkins 2005). The utilization of the NTN model provides a useful platform for investigating the pathogenesis of glomerulonephritis and exploring potential therapeutic strategies.

## Establishment of the NTN model

### Preparation and characteristics of nephrotoxic serum

High-quality nephrotoxic serum (NTS) is a key factor for the successful establishment of NTN models. Heterologous animals such as rats, rabbits, or sheep are commonly selected for the preparation of NTS (Robson et al. 2001; Kaneko et al. 2006; Kitagawa et al. 2019). In early studies, whole kidney tissue suspensions were used to immunize heterologous animals, as homologous anti-renal serum lacks nephrotoxic components (Smadel n.d). However, the composition of whole-kidney extracts is complex and has low specificity, resulting in inconsistent efficacy in inducing nephritis. Subsequent adsorption experiments demonstrated that NTS primarily reacts with glomerular tissue, suggesting that the key antigens responsible for inducing anti-renal serum production originate in the glomerulus (Solomon and Gardella 1949; Morley and Wheeler 1985). Therefore, the preparation method was modified to use crude glomerular extracts or their GBM as the immunogen (Morley and Wheeler 1985; Fau et al. (Salant n.d.). Now, the standard approach requires immunizing donor animals (e.g., sheep) with purified GBM components that are homologous to the target species of the nephritis model. Following complement inactivation and red blood cell adsorption treatment, the resulting antiserum is collected and administered to model animals to induce nephritis (Madaio et al. 1984). With the progressive identification of specific antigens within NTS, monoclonal antibodies or antisera targeting defined components, such as α3β1 integrin, type IV collagen, laminin, and aminopeptidase A, are increasingly used as alternatives to traditional NTS (Chugh et al. 2001; O'Meara et al. 1992; Hara et al. 1991; Shichinohe et al. 2002).

### Genetic and species susceptibility in NTN models

Currently, mice and rats are widely employed in establishing NTN models, but different strains exhibit different sensitivities to the NTS. In fact, early in the application of the NTN model, researchers have investigated the impact of animal strain and sex on NTN model establishment (Smadel and Farr 1937). In mice, susceptibility to NTN varies among inbred strains and strongly influences the severity of inflammation (Huang et al. 1997; Xie et al. 2004, 2008). For example, although C57BL/6, 129/sv and CD1 mice are easily inducible for NTN, C57BL/6 mice exhibit greater susceptibility to glomerular inflammation than 129/Sv mice do, and CD1 mice are more susceptible than the C57BL/6 strain (Robson et al. 2003; Ougaard et al. 2018a). Since differences in the systematic bias of background genes significantly affect disease susceptibility in mice, the genetic background should be carefully considered when using genetically engineered mice to establish an NTN model.

In addition to genetic background, sex is another factor that should be considered when establishing the NTN model. Some studies have indicated that female NTN mice, especially CD1 mice, display increased urinary albumin excretion during prolonged monitoring (more than 5 months) (Ougaard et al. 2018a); however, young male mice are more sensitive than females (Garcia et al. 2023). Thus, many studies still use male mice to establish NTN models, possibly because their observation periods are relatively short. Besides, according to the literature, mice older than 8 weeks are relatively suitable for model establishment, with some studies even utilizing 12-week-old mice (Pace et al. 2021; Haddad et al. 2023; Alli et al. 2023; Casalena et al. 2014; Nagayama et al. 2014).

Wistar-Kytot (WKY) and Sprague–Dawley (SD) rats are two rat strains commonly used to establish NTN models (Cai et al. 2015; Kanno et al. 1998). WKY rats exhibit high susceptibility to immune-mediated renal injury and are prone to develop in situ immune complexes following the administration of NTS, which can induce typical glomerular crescentic lesions (Masayuki et al. 2009). SD rats are also widely used due to their stable immune responses and relatively high success rate in model induction. The pathological features of the NTN model in SD rats resemble those that are observed in human crescentic nephritis, including the presence of proteinuria, glomerular basement membrane damage, and inflammatory cell infiltration (D B W, R M, S N Z, A J R. 1985).

### Experimental approaches for NTN induction

**Fig. 1:**
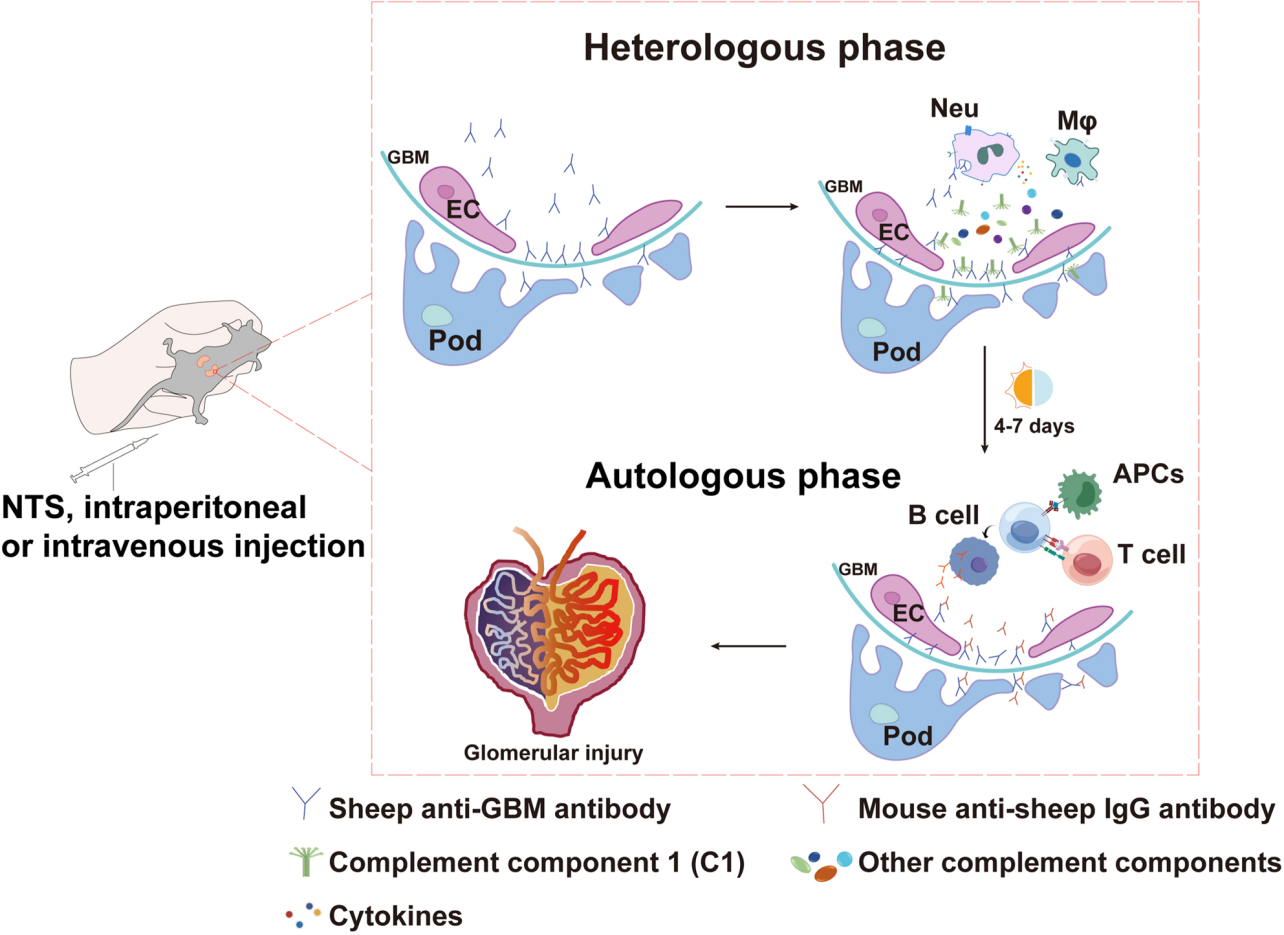
Pathogenic mechanisms of NTN model. During the heterologous phase, nephrotoxic serum derived from a different species (sheep, as shown here) and containing anti-glomerular basement membrane (GBM) antibodies is injected into mice. These antibodies rapidly bind to and deposit along the GBM, while a fraction of sheep IgG also binds to the surface of glomerular cells, including podocytes and glomerular endothelial cells. Deposition of heterologous antibodies leads to the formation of immune complexes and activation of the complement cascade, which promotes the recruitment of phagocytes such as neutrophils and macrophages. These cells phagocytose heterologous antibodies and release pro-inflammatory mediators, resulting in local glomerular inflammation. As the disease progresses, sheep IgG is taken up by professional antigen-presenting cells (APCs) or other APCs and presented to B cells. With the help of CD4⁺ T cells, B cells differentiate into plasma cells and produce mouse autoantibodies against sheep IgG, marking the autologous phase. These autoantibodies bind to sheep IgG and may deposit at additional sites within the glomerulus, forming larger immune complexes and thereby amplifying glomerular injury. The pattern shown in the figure was created with the help of BioGDP.com (Jiang et al. 2025). *NTS* Nephrotoxic serum, *GBM* Glomerular basement membrane, *EC* Vascular endothelial cell, *Pod* Podocyte, *Neu* Neutrophil, *Mφ* Macrophage, *APC* Antigen-presenting cell

The NTS can be delivered through the intraperitoneal or intravenous (through the tail vein or retroorbital) routes in experimental animals (Butt et al. 2022; Tesch et al. 2021; Hackl et al. 2023). After NTS administration, experimental animals undergo two phases of the immune response: the heterologous phase and the autologous phase (Fig. 1). Following NTS injection, the heterologous antibodies within NTS rapidly deposit in the GBM for antigen–antibody reactions (Hoppe and Vielhauer 2014). The heterologous phase triggered by heterologous antibodies occurred in GBM is an acute immune response. Approximately 4–7 days after NTS injection, the chronic autologous phase begins, featuring adaptive immune responses mainly against heterologous immunoglobulins. Many antibodies that anti-heterologous immunoglobulin are produced by B cells of experimental animals, which further leads to glomerular injury (Johnson and Zager 2025; Thurman et al. 2012; Takahashi et al. 1990; Artinger et al. 2017). On the basis of these two phases, the induction methods of the NTN model can be categorized into two types: accelerated and nonaccelerated models. For the accelerated model, prior to immunization, adjuvants are administered first, followed by the administration of NTS 5–8 days later (Kasinath et al. 2019). The use of adjuvants shortens or eliminates the heterologous phase (Robson et al. 2001). In contrast, nonaccelerated models are generated via direct injection of NTS without prior immunization, resulting in relatively long-term and mild symptoms (Madaio et al. 2019; Chen et al. 2018; Schmidt et al. 2021).

Notably, the diagnostic criteria for classifying NTN models as acute/chronic or mild/moderate/severe vary among studies. An acute NTN model induced by the accelerated method can be maintained for up to 3 weeks before termination (Topham et al. 1999; Lloyd et al. 1997), whereas another NTN model established via the nonaccelerated method can be harvested at 24–72 h, with dramatic proteinuria and podocyte injury observed (George et al. 2012). Moreover, some studies have associated low, medium, and high doses of NTS with mild, moderate, and severe pathological damage, respectively (Madaio et al. 2019). Nevertheless, another study showed that even high doses of NTS may not cause glomerular crescents even there was significant proteinuria (D'Souza et al. 2013). Specifically, the dosage of NTS varies considerably across different studies, which may be attributed to variations in efficacy among serum batches. Studies indicate that although nephrotoxicity can be demonstrated in vivo, the severity of NTS-induced injury does not directly correlate with the titer of precipitins against kidney extracts in NTS (J E S. 1936). Since the capacity of different NTS batches to induce renal injury varies considerably, it is worthwhile to perform pre-experiment to establish a more standardized and stable experimental process.

## Pathological characteristics of the NTN model and their clinical relevance

### GBM-associated antibody deposition

Abundant antibody deposition along the GBM represents a hallmark pathological feature of the NTN model. NTS consists predominantly of anti-GBM antibodies, along with smaller amounts of antibodies directed against other glomerular cellular components. Following administration, anti-GBM antibodies rapidly deposit along the GBM, with prominent linear heterologous antibody deposition detectable as early as 1 h after NTS injection (Madaio et al. 1984). During the subsequent autologous phase, newly generated endogenous antibodies also predominantly localize to the GBM (Madaio et al. 1984). The GBM is a specialized structure that is particularly susceptible to antibody and immune complex deposition, in part due to fixed anionic sites within the glomerular capillary wall that promote antigen localization and facilitate antibody deposition in the subepithelial space (Xu et al. 2024; Anders et al. 2023). In this context, the GBM plays a critical role in the pathogenesis of immunological renal diseases, serving either as a primary antigen targeted by autoantibodies or as a site for immune complex deposition (Balasubramaniyan et al. 2017).

Antibody deposition within the GBM represents a key initiating event in various forms of glomerular injury. Clinically, anti-GBM disease is a rare but life-threatening autoimmune disorder caused by autoantibodies directed against GBM antigens (Francesco et al. 2023; Min et al. 2009; Zhang et al. 2023) and is among the few human autoimmune diseases with well-defined pathogenic autoantigens (Min et al. 2009). The antibodies deposited within the GBM is highly specific (Balasubramaniyan et al. 2017; Omid et al. 2018; Kimia et al. 2023). These antibodies predominantly target the noncollagenous domain 1 (NC1) of the α3 chain of type IV collagen (α3[IV]) and, less commonly, the α4(IV) or α5(IV) chains, which together form a triple-helical structure within the GBM (Omid et al. 2018; Kimia et al. 2023). Structural alterations in the hexameric configuration of α3(IV) NC1 expose cryptic epitopes, thereby initiating an autoimmune response and resulting in linear deposition of antibodies-predominantly IgG-along the GBM (Omid et al. 2018; Kimia et al. 2023). Beyond anti-GBM disease, antibody deposition within the GBM is also a defining feature of several other renal pathologies. In primary membranous nephropathy (MN), approximately 70–80% of cases exhibit subepithelial immune complex deposition composed of phospholipase A2 receptor (PLA2R) or thrombospondin type-1 domain-containing 7 A (THSD7A) and their corresponding autoantibodies, a pattern considered pathognomonic for the disease (Sanjeev et al. 2019). Notably, in MN, immunofluorescence staining for IgG showed a bright granular deposition along GBM (Sethi 2021). In addition, monoclonal immunoglobulin deposition disease (MIDD), a rare condition accounting for less than 1% of renal biopsy diagnoses, is characterized by the deposition of monoclonal immunoglobulins-light chains, heavy chains, or both-along basement membranes, including the GBM, ultimately leading to progressive renal dysfunction (Michael et al. 2020).

### Development of severe proteinuria

Severe proteinuria is observed in the NTN model as early as the initial phase after NTS administration, e.g. within 4 h (Arif et al. 2019a). The rapid deposition of antibodies along the GBM is the primary mechanism underlying the early onset of severe proteinuria in this model. On the one hand, antibody deposition rapidly disrupts the surface charge and structural integrity of the GBM, leading to impairment of both the structural framework and the charge-selective barrier (Menon et al. 2012). On the other hand, activation of the complement system and recruitment of inflammatory cells further damage podocytes and glomerular endothelial cells on both sides of the basement membrane, resulting in additional disruption of the glomerular filtration barrier (Hoppe and Vielhauer 2014; Rodriguez et al. 2007; Saito et al. 2025). In addition, although the antigens that trigger its production are composed primarily of basement membrane components, they may also include cellular elements such as podocytes and vascular endothelial cells (Chugh et al. 2001; O'Meara et al. 1992). Therefore, a minor fraction of antibodies in NTS directed against glomerular cellular components may directly injure glomerular cells. The presence of proteinuria, podocyte injury, glomerular endothelial cell damage, and subsequent renal fibrosis highlights the diverse pathological features of the NTN model and broadens its scope of application.

Some studies suggest that proteinuria in the NTN model exhibits a transient peak, followed by a gradual return to near-normal levels (Ougaard et al. 2018b; Chung et al. 2020; Siegerist et al. 2024), whereas others report persistent and stable high-level proteinuria (Tesch et al. 1999; Abbad et al. 2024; Sakurai et al. 1997). These differences may be related to the strength of the autologous immune response. For example, in certain cases, proteinuria reaches a peak within one day and rapidly declines, becoming largely resolved by day 5, but with no detectable glomerular deposition of mouse IgG throughout the 1–21-day observation period (Chung et al. 2020). Regardless of these variations, the development of proteinuria represents the minimal criterion for successful induction of the NTN model and remains one of the most accessible and reliable indicators for monitoring disease progression.

### Contribution of innate and adaptive immune cells

In the pathogenesis of the NTN model, the extent of immune cell infiltration has been shown to correlate with both clinical severity and pathological damage in animal models and human disease (Panzer et al. 2007). Recent studies have reported that multiple immune cell populations including neutrophils (Saito et al. 2025), T lymphocytes (Mooslechner et al. 2022), B lymphocytes (Li et al. 2023a), dendritic cells (Miao et al. 2025) and macrophages (Tatsumoto et al. 2023), play key pathogenic or protective roles through mechanisms such as direct infiltration into renal tissue, regulation of immune complex clearance, and participation in inflammatory responses. The immune cell types involved in the NTN model have been briefly summarized in Table 1.

**Table 1:** Roles of immune cell subsets in the NTN model

Immune cells involved In the NTN model	Function	References
Neutrophils	The infiltration of this characteristic marker cell, via complement activation, initiates inflammation and typifies the morphology of glomerular injury	(Khalighi and Chang 2021)
	Enhancing immune complex clearance via receptors (e.g., C3bR CR1/2 and FcγRII/III) to reduce deposition, thereby alleviating injury and protecting the kidneys	(Saito et al. 2022)
Macrophages	The polarization state of infiltrating macrophages determines their significant roles in renal damage, specifically, M1 macrophages exacerbate glomerulonephritis through the production of proinflammatory cytokines such as TNF-α	(Katsuyama et al. 2021; Wen et al. 2019; Chen et al. 2009)
T Lymphocytes	Modulating the Th1/Th17/Treg balance (pro-/antiinflammatory) via the TNF-α/NF-κB (IKK-2/NEMO) pathway and SRSF1	(Artinger et al. 2017; Katsuyama et al. 2021; Wen et al. 2019; Guo et al. 2019)
	Promoting inflammation via STAT3 (enhances autoantibody/infiltration)	(Yoshida et al. 2019)
	Moleculing SRSF1 (via RhoH) and Tim3 negatively regulate T-cell activation to protect kidneys by suppressing excessive inflammation; their loss or blockade exacerbates nephritis	(Katsuyama et al. 2021; Schroll et al. 2010)
	External interventions work via T cells: GLP-1 agonists inhibit T-cell proliferation; low-dose IL-15 reduces renal injury dependent on CD8 + T cells	(Mooslechner et al. 2022; Moschovaki Filippidou et al. 2020)
B Lymphocytes	Producing anti-gbm antibodies (targeting the type IV collagen α3 (IV) NC1 domain) which deposited in GBM in a linear pattern, directly triggering anti-GBM disease/Goodpasture disease and leading to cDN	(Chen et al. 2009; Kazemzadeh et al. 2023; Reggiani et al. 2023; Sadeghi-Alavijeh et al. 2018; Chen et al. 2010; Yang et al. 2007; Salama et al. 2002)
	Producing anti-PLA2R/THSD7A antibodies, which forming immune complexes with target antigens and deposit in GBM, mediating primary membranous nephropathy (MN)	(Sethi et al. 2019)
	Generating monoclonal immunoglobulins (light chains/heavy chains), deposits them in GBM, mediates monoclonal immunoglobulin deposition disease (MIDD) and monoclonal IgG deposition proliferative glomerulonephritis	(Turner et al. 2020; Fujita et al. 2012)

Glomerulonephritis is driven by an inflammatory response to glomerular deposition of immunoglobulins directed against endogenous or exogenous antigens, with neutrophils representing the predominant inflammatory cell population at the early stage of disease (Inoue et al. 2022). Among the infiltrating leukocytes, neutrophils together with macrophages function as the principal effector cells mediating tissue injury in glomerulonephritis (Tatsumoto et al. 2023). Studies showed that in the NTN model, neutrophils are rapidly recruited to the glomeruli within a few hours following nephrotoxic serum challenge (Schrijver et al. 1990), preceding the subsequent infiltration of macrophages, which typically occurs around day 7 (Du et al. 2016), thereby underscoring the central role of neutrophils in initiating glomerular inflammation. Beyond their role as initiators and amplifiers of inflammation, neutrophils may also contribute to the attenuation of glomerular injury by promoting immune complex clearance via specific surface receptors. For instance, with overexpression of angiotensin-converting enzyme (ACE) in neutrophils promotes the uptake and clearance of immune complexes formed locally within the glomeruli (Suguru et al. 2022), and thereby ameliorating glomerular injury.

In addition to neutrophils, other immune cell populations also play crucial roles in the progression of NTN model. Both clinical observations and experimental studies demonstrated marked leukocyte infiltration-particularly macrophages and CD4⁺ and CD8⁺ T cells-in experimental glomerulonephritis models as well as in renal biopsies from patients with glomerulonephritis (Mooslechner et al. 2022; Sakatsume et al. 2001; Xie et al. 2024; Wen et al. 2020; Antonelou et al. 2020). These infiltrating leukocytes together with neutrophils constitute the predominant inflammatory cell population in many forms of glomerulonephritis (Muhammad 2014). Notably, in NTN models and other glomerular injury models, the infiltration of inflammatory cells usually occurs in the renal interstitium, particularly in the periglomerular region, rather than within the glomerulus itself (Chen et al. 2018; Janssen et al. 1998; Vasilopoulou et al. 2016; Paust et al. 2011; Lim 2014; Yang et al. 2020), One proposed mechanism is that, in the NTN model, complement activation and glomerular injury generate diffusible signals that promote inflammatory cell recruitment to the renal interstitium. When Bowman’s capsule remains intact, recruited cells accumulate periglomerularly and interact with interstitial endothelial cells, tubular epithelial cells, and fibroblasts, inducing proinflammatory mediator and cytokine release that drives glomerulosclerosis and interstitial fibrosis. Upon capsule rupture, infiltrating leukocytes directly enter the glomeruli and stimulate glomerular cells (Muhammad 2014; Lan et al. 1992).

### Injury to renal resident cells

In the NTN model, additional pathogenic mechanisms contribute to injury of glomerular resident cells and are not exclusively dependent on immune complex-mediated pathways. NTS is generated by immunization with crude glomerular extracts (Salant and Cybulsky 1988). Although the antigens responsible for NTS production consist predominantly of GBM components, they may also include cellular antigens derived from glomerular resident cells (O'Meara et al. 1992; Mendrick et al. 1983; Orikasa et al. 1988). Notably, purified individual antibody fractions isolated from NTS are sufficient to induce severe proteinuria. Further characterization of NTS has revealed that antibodies targeting specific glomerular cell surface proteins, such as aminopeptidase A, may play a critical role in proteinuria induction (Chugh et al. 2001). Consistent with this concept, a study from Kyorin University demonstrated that immunization with the podocyte foot process protein Crb2 elicited anti-Crb2 autoantibodies and resulted in severe proteinuria with features characteristic of primary nephrotic syndrome (Hada et al. 2022). Moreover, Direct exposure to NTS induces podocyte injury, which appears to be mediated by anti-podocyte antibodies present in the serum, including anti-nephrin antibodies (Arif et al. 2019a; Haddad and Blaine 2025; Huang et al. 2025), and the complement system cloud also be activated by NTS induction in vitro (Haddad and Blaine 2025). Supporting this notion, early phases of NTS-induced nephritis in vivo exhibit a component of noninflammatory injury and does not appear to result from antigen–antibody reactions at the GBM (Chugh et al. 2001). Besides, antibodies against certain glomerular components may be not necessary for inducing proteinuria. For instance, although NTS contains antibodies against the noncollagenous domain 1 (NC1) of the α3 chain of type IV collagen, depletion of these antibodies does not abolish the proteinuric effect of NTS, suggesting that they are not essential mediators (Chugh et al. 2001).

Collectively, these findings indicate that renal injury encompasses a continuum ranging from immune complex-mediated damage to non-immune complex-dependent mechanisms affecting glomerular endothelial cells, epithelial cells, and tubular epithelial cells (Gavin et al. 2008). While immune cells are traditionally regarded as the primary source of inflammatory mediators, accumulating evidence demonstrates that nonimmune cells, including glomerular endothelial cells, mesangial cells, and podocytes, actively participate in renal inflammation by producing cytokines and chemokines, thereby directly or indirectly aggravating glomerular injury (Yongqing et al. 2025; Medina Rangel et al. 2021). These observations underscore that nonimmune factors play a substantial role in NTS-induced glomerular injury via intricate cellular and molecular networks.

## Applications of the NTN model in renal disease research

At present, the NTN model has been widely used to investigate glomerulonephritis, lupus nephritis, focal segmental glomerulosclerosis, and renal fibrosis (Li et al. 2023a; Umeda et al. 2025; Lazareth et al. 2025; Bronstein et al. 2025). Besides, this model has also been applied to investigate renal resident cell injury, including podocyte, parietal epithelial cells, and tubular epithelial cell involvement (Bronstein et al. 2025; Siegerist et al. 2025; Chen et al. 2025; Chung et al. 2021; Lopatko Fagerstrom et al. 2019; Zhen et al. 2018). Representative studies employing these models and the specific pathological features they address are summarized in Table 2. Glomerulonephritis is defined by damage to the structure and function of the glomerulus and results from a combination of primary and secondary pathogenic factors. As the disease progresses, fibrosis often develops and can eventually lead to end-stage renal disease. Although immune-mediated mechanisms predominate during disease development, nonimmune factors also play a substantial and often underappreciated role. In experimental studies, the NTN model displays multiple key features of glomerular injury, making it a useful tool for modeling diverse glomerular diseases and injury-associated pathological states.

**Table 2:** Renal diseases studied using the NTN model

Disease	Target cells
Crescentic glomerulonephritis	Neutrophils, macrophage, T cells, kidney lymph node-resident fibroblastic reticular cells (Kasinath et al. 2019; Saito et al. 2025; Tatsumoto et al. 2023; Guan et al. 2023)
Lupus nephritis	B cells, myeloid cells, tubular epithelial cell, CD11b^+^ cells (Kitagawa et al. 2019; Alli et al. 2023; Chalmers et al. 2021, 2019)
Acute glomerulonephritis	Muller et al. 2019)
Proteinuric kidney disease	Podocytes (Guo et al. 2024)
Podocytopathy	Podocytes (Guo et al. 2024)
FSGS	Parietal epithelial cells (Bronstein et al. 2025)
Vasculitis	Glomerular endothelial cells (Lopatko Fagerstrom et al. 2019)
Kidney fibrosis	Macrophage, cortical tubular epithelial cells, T cells/macrophages, podocytes (Wen et al. 2019; Kim et al. 2016; Tampe et al. 2021; Arif et al. 2019b; Inoue et al. 2015)
Renal tubular ammoniagenesis	(Johnson and Zager 2025)

### Immune-mediated glomerulonephritis

The NTN model is widely used for mechanistic studies and preclinical drug evaluation in glomerulonephritis because it reproduces key pathological features of human antibody-mediated glomerulonephritis (Michael P et al. 2019). In therapeutic development, the NTS nephritis model in WKY rats reliably induces crescentic glomerulonephritis (cGN) with low doses of NTS and is accompanied by a gradual decline in renal function over several weeks, making it a robust platform for screening antinephritic agents (K et al. 1998). Using this model, prostaglandin E2 (PGE2) has been shown to improve blood urea nitrogen levels, reduce proteinuria, and ameliorate histopathological injury in NTS-induced acute nephritis (Nino et al. 2013). Similarly, recombinant human thrombomodulin (rhTM) attenuates renal fibrosis in cGN, supporting its potential therapeutic value (Nobuhiro et al. 2020). Beyond drug evaluation, the NTN model has been instrumental in dissecting molecular mechanisms underlying glomerulonephritis. Retinoic acid (RA) protects podocytes in cDN through activation of podocyte-specific retinoic acid receptor-α (RARα) (Yan et al. 2017). Myeloid-specific deletion of myeloid cell leukemia 1 (Mcl-1) reduces histological injury, proteinuria, and inflammatory responses (Yusuke et al. 2020), and podocyte-restricted signal transducers and activators of transcription 3 (STAT3) deficiency limits crescent formation and preserves renal function (Yan et al. 2013). Nitric oxide (NO) exerts a protective effect in NTS-induced glomerulonephritis (Roland and Karen 2002). In contrast, the Axl receptor tyrosine kinase promotes disease progression by enhancing mesangial cell proliferation, and its inhibition alleviates glomerulonephritis severity (Yuxuan et al. 2021). Indoleamine 2,3-dioxygenase (IDO) has also been identified as a negative regulator of NTN progression (Weiping et al. 2009). In addition, 17β-estradiol may modulate autoimmune nephritis by regulating VCAM-1 expression in mesangial cells (Neelakshi and Roberto 2015).

In the context of immune and inflammatory regulation, tumor necrosis factor (TNF) derived from local immune deposits activates proinflammatory signaling pathways in glomerular endothelial cells and podocytes (Michael P et al. 2019). MER receptor tyrosine kinase regulates immune-mediated glomerulonephritis by promoting apoptotic cell clearance and modulating immune responses (Wen-Hai et al. 2010). Regulatory T (Treg) cells play a critical role in the resolution of cDN, and therapeutic approaches that expand Treg populations may help limit renal injury (Yoshitsugu et al. 2011). Notably, prophylactic inhibition of IκB kinase β (IKK2) suppresses dendritic cell and T helper cell activation and alleviates NTS-induced glomerulonephritis; however, therapeutic inhibition of IKK2 can exacerbate disease, likely due to the concurrent depletion of Treg cells (Janine et al. 2015).

Beyond immune regulation, the NTN model has been widely used to explore disease susceptibility and identify potential biomarkers. Decay-accelerating factor (DAF)-deficient mice display increased sensitivity to NTS and develop glomerulonephritis even when exposed to subnephritogenic doses of NTS (H et al. 2001). Similarly, DAF/Crry double-deficient mice exhibit more severe proteinuria in response to NTS, despite reduced systemic complement activity (Takashi et al. 2006).

### Lupus nephritis

The NTN model is a valuable experimental tool for studying the pathogenesis of lupus nephritis (LN), evaluating therapeutic strategies, and elucidating underlying mechanisms (Feng et al. 2023). In studies of cell migration and origins, experiments using MRL/lpr lupus mice and the NTN model revealed that group 3 innate lymphoid cells (ILC3s) accumulating in the kidneys primarily originate from the intestinal tract. This finding provides novel insights into the sources of locally infiltrating immune cells during LN (Yajuan et al. 2015). In therapeutic exploration, mesenchymal stem cells (MSCs) transduced with human tissue kallikrein 1 (hKLK1) reduce renal macrophage and T cell infiltration, in part by suppressing of inflammatory cytokine expression, and exhibit enhanced resistance to oxidative stress-induced apoptosis. These properties suggest their potential as kidney-targeted gene delivery vehicles for modulating inflammation and oxidative stress in LN (Yajuan et al. 2013). Similarly, T-cell-specific silencing of STAT3 impairs the capacity of T cell to support autoantibody production by B cells and limit tissue infiltration, highlighting a promising therapeutic approach for LN (N et al. 2019). Dietary intervention studies further indicate that the effects of therapeutic strategies may be context dependent. Although fish oil supplementation preserves renal function in spontaneous lupus models, it exacerbates renal injury in an accelerated NTS nephritis model, underscoring important differences among experimental systems (L S, M M, H H, A S, D S, N G,, et al. 1990). From an immunological perspective, interferon-γ (IFN-γ) produced by CD4⁺ T helper 1 (Th1) cells plays a key role in driving inflammation in systemic lupus erythematosus (SLE) and LN (Takayuki et al. 2020). This pathway partially overlaps with immune-mediated inflammatory mechanisms observed in the NTN model, providing a rationale for comparative and translational studies across these models.

When integrated with spontaneous lupus models, the NTN model contributes to elucidating immune cell origins in LN, assessing novel cellular and gene-based therapies, and advancing the understanding of immune regulatory pathways, thereby supporting both basic research and translational applications. However, the NTN model cannot fully substitute for spontaneous LN models. LN represents the renal manifestation of SLE and is driven by systemic autoimmune responses against autoantigens such as double-stranded DNA (Liu et al. 2026). In contrast, the NTN model relies on exogenously administered antibodies and does not capture the systemic autoimmune milieu characteristic of SLE. Future model refinement may involve introducing genetic susceptibility to autoimmunity, combined with repeated low-dose antiserum administration and relevant comorbid conditions, such as hypertension, to better mimic chronic disease progression. Integrating the strengths of multiple experimental approaches may ultimately yield models that more faithfully reflect the complex pathophysiology of LN.

### Glomerulosclerosis

The NTN model has been widely used to investigate the mechanisms underlying immune-mediated glomerular injury and progressive glomerulosclerosis. Importantly, this model recapitulates several key cellular events implicated in the pathogenesis of focal segmental glomerulosclerosis (FSGS), including rapid podocyte loss followed by activation and proliferation of parietal epithelial cells (PECs) (Shankland et al. 2014). In the NTN model, podocyte injury occurs at a very early stage, the structural and functional damage of podocytes can be observed within 24 h after injection of NTS (George et al. 2012). These findings support the concept that podocyte depletion represents a triggering event in various forms of glomerulonephritis and in FSGS (Cunanan et al. 2025; Kim et al. 2016; Li et al. 2023b). Both in vivo and in vitro experiments demonstrate that NTS can directly induce podocyte injury, an effect attributed to the presence of anti-nephrin antibodies in the serum (Haddad and Blaine 2025). Nephrin is a core component of the slit diaphragm and is essential for maintaining the integrity of the glomerular filtration barrier (Wang et al. 2021). Loss of nephrin expression leads to proteinuria, a hallmark of podocyte injury and nephrotic syndrome (Huh et al. 2002). Autoantibodies against nephrin have been detected in patients with minimal change disease and recurrent FSGS (Shu et al. 2025; Hengel et al. 2025). Consistently, NTS contains anti-nephrin antibodies and induces injury in cultured murine podocytes through direct antibody binding, with involvement of the complement system (Haddad and Blaine 2025). These observations provide a mechanistic explanation for the early onset of proteinuria and podocyte dysfunction observed in the NTN model.

Following podocyte loss, aberrant activation and proliferation of PECs emerge as a prominent pathological feature that drive crescent formation and ultimately lead to glomerular sclerosis. This sequence of events has been documented across multiple glomerular disease subtypes (Bronstein et al. 2025; Miesen et al. 2022; Cruzado et al. 2024). Although parietal epithelial cell activation occurs more rapidly and extensively in cGN, the underlying epithelial activation pathways are largely shared between cGN and FSGS, differing mainly in the speed and intensity of the response (Lazareth et al. 2025). These shared features have prompted increasing use of the NTN model to explore non-immune mechanisms of glomerular injury and scarring that are also relevant to FSGS.

Clinically relevant disease features in the NTN model include the rapid onset of proteinuria, which may reach a peak and subsequently enter a plateau phase or partially resolve. Histopathological analyses performed during both phases reveal prominent crescent formation, FSGS-like lesions, tubular injury and interstitial inflammation. In longer-term studies, NTS-induced glomerulonephritis progresses over 4–6 weeks to overt glomerulosclerosis and periglomerular fibrosis, even at time points when proteinuria has returned to near-normal levels (Chung et al. 2020). Together, these findings indicate that glomerular injury continues to accumulate over time despite apparent improvement in proteinuria. At experimental endpoints, the NTN model is characterized by intraglomerular extracellular matrix deposition. Collectively, these pathological features closely resemble those observed in human glomerulonephritis and progressive glomerulosclerosis (Bollee et al. 2011).

Accordingly, the NTN model has proven valuable for studying molecular pathways that contribute to glomerulosclerosis. Genetic and pharmacological interventions targeting these pathways have established key roles in disease progression. For example, deletion of YAP results in more severe and sustained proteinuria and exacerbated glomerulosclerosis following NTS injury (Chung et al. 2020). By contrast, deletion of canonical transient receptor potential-6 (TRPC6) attenuates glomerulosclerosis in the NTN model (Kim et al. 2019). In addition, bromodomain and extra-terminal domain (BET) inhibitors exert renoprotective effects and reduce glomerulosclerotic lesions (Morgado-Pascual et al. 2022). Dysregulation of the KLF4-STAT3 signaling axis in podocytes has also been shown to promote abnormal proliferation of glomerular epithelial cells in NTN model and patients, further highlighting shared pathogenic mechanisms across these diseases (Estrada et al. 2018).

Despite these similarities, several important limitations should be considered when applying findings from the NTN model to FSGS and nephrotic syndrome. In the NTN model, the initial injury is triggered by antigen–antibody complex formation, which is fundamentally different from the primary podocyte injury that underlies most cases of primary FSGS. Consequently, although the NTN model reliably reproduces downstream events such as podocyte loss and epithelial cell dysfunction, it may not fully reflect the earliest disease-initiating processes in idiopathic FSGS. In addition, studies using the NTN model to examine FSGS-like lesions have mainly focused on histological and molecular outcomes, whereas comprehensive evaluation of kidney function, body weight changes and persistent nephrotic-range proteinuria is often lacking. As a result, the extent to which the NTN model captures the full clinical spectrum of nephrotic syndrome remains unclear.

In summary, the NTN model provides a valuable experimental system for studying podocyte injury-driven glomerulosclerosis and epithelial pathways relevant to FSGS. However, its immune-based mode of disease initiation, together with limited assessment of functional and systemic features, restricts its usefulness as a model of primary FSGS and nephrotic syndrome. Future studies that link kidney function to pathological injury, track phenotypic changes over time, and explore the underlying mechanisms will be especially important for more clearly defining the strengths and limitations of the NTN model in FSGS and nephrotic syndrome research.

### Other applications, including renal fibrosis and tubular injury

The NTN model serves not only as a system for studying glomerular injury but also as a valuable tool for investigating the mechanisms underlying pathological processes such as renal fibrosis and tubular damage. Several studies have elucidated the contributions of various factors to these processes.

In the NTN model, induction of isolated cDN leads to chronic glomerular injury, resulting in prominent focal fibrosis adjacent to atrophic tubules (Désirée et al. 2021). Macrophages and T lymphocyte-derived factors play pivotal roles in the pathogenesis of fibrosis. For example, macrophage-specific deletion of TNF-α significantly reduces both glomerular and tubular damage and attenuates renal fibrosis (Yi et al. 2019). Similarly, T lymphocyte-derived TNF-α contributes to the attenuation of renal injury and fibrosis in nephrotoxic nephritis (Yi et al. 2019). During the fibrotic phase (days 14–35) of cDN, the administration of a selective c-fms kinase inhibitor (fms-I) modulates macrophage activity. By day 14 post-NTN induction, rats already exhibit significant macrophage infiltration, tubulointerstitial injury, and renal dysfunction (Yingjie et al. 2013). Furthermore, treatment with an TNF-like weak (TWEAK) inducer of apoptosis monoclonal antibody markedly reduces glomerular immunoglobulin deposition, macrophage infiltration, and tubulointerstitial fibrosis, accompanied by improvements in proteinuria and renal histopathology in NTN mice (Yumin et al. 2012).

In the NTN model, tubular injury often occurs alongside fibrotic changes. Macrophage infiltration and TWEAK-driven inflammatory responses jointly promote tubulointerstitial fibrosis and structural damage to renal tubules (Yumin et al. 2012). Activation of the TNF-α signaling pathway promotes both fibrogenesis and tubular damage, and its pharmacological inhibition has been shown to ameliorate both processes (Yi et al. 2019). Furthermore, focal fibrosis resulting from chronic glomerular injury often localizes adjacent to atrophic tubules, highlighting a pathogenic interplay between glomerular damage, tubular atrophy, and fibrotic remodeling (Désirée et al. 2021).

## Conclusion

The nephrotoxic nephritis (NTN) model remains a robust and widely used platform for studying antibody-mediated glomerular injury, offering valuable insights into key pathogenic processes such as antibody deposition, complement activation, and immune cell recruitment. Despite its strengths, the model has limitations, including its inability to fully recapitulate chronic kidney disease progression and complex immune mechanisms seen in conditions like lupus nephritis (Table 3). Variability in experimental conditions may also affect reproducibility. Nevertheless, when combined with emerging approaches such as genetic models and organ-based technologies, the NTN model will continue to play an important role in advancing mechanistic understanding and therapeutic development in kidney diseases.

**Table 3:** Strengths and limitations of the NTN model

Advantages of the NTN model	Limitations of the NTN model
Prominent antibody deposition• The NTN model is characterized by robust glomerular antibody deposition, including substantial endogenous autoantibody accumulation. This feature enables effective modeling of glomerulonephritis types in which antigen–antibody complex deposition constitutes a central pathological hallmark	Exogenously induced disease initiation • Disease induction in the NTN model relies on the exogenous administration of anti-GBM antibodies, with rapid antibody deposition initiating injury at the basement membrane. In contrast, human glomerulonephritis often arises from complex mechanisms involving genetic susceptibility and dysregulated immune tolerance, including the initial generation of autoantibodies, which are not represented in this model
Marked complement activation • Deposition of both heterologous IgG and endogenous IgG leads to marked activation of the complement system. This property makes the NTN model a useful experimental tool for investigating the pathogenic mechanisms of complement activation in glomerulonephritis	Restricted representation of antibody classes• In the NTN model, antibody deposition assessment primarily focuses on IgG. However, human glomerulonephritis is often characterized by IgA deposition or specific IgG subclasses. Consequently, the NTN model fails to adequately recapitulate glomerular diseases defined by these distinct immunoglobulin deposition patterns
Immune cell-renal cell interactions • The involvement of infiltrating immune cells alongside injury to intrinsic renal cells highlights critical interactions between inflammatory cells and resident glomerular cells. Such interactions represent a fundamental and distinctive aspect of glomerulonephritis pathogenesis and can be effectively studied using this model	Acute and rapid disease course• NTN typically manifests as an acute and rapidly progressive injury, with marked proteinuria developing over a short period. This contrasts sharply with the chronic and often insidious progression of many human glomerulonephritis
Targeted injury to podocytes and endothelial cells• Nephrotoxic serum used to induce NTN contains antibodies directed against podocyte and glomerular endothelial cell components, allowing relatively independent injury to these cell types. This is well suited for investigating mechanisms of podocyte and endothelial cell injury	Limited reproducibility across experimental settings• Responses to nephrotoxic serum vary among different animal strains. In addition, variability in serum potency between batches and differences in induction protocols across laboratories hinder the standardization of NTN model
Clear and distinct immunological phases • The NTN model proceeds through two well-defined stages: a heterologous phase, characterized by the direct binding of injected antibodies, and an autologous phase, driven by the host immune response against the foreign antibodies. This temporal separation allows different components of the immune response to be examined independently	Restricted immune pathway representation• The NTN model predominantly activates complement-dependent and Fc receptor-mediated inflammatory pathways. In human glomerulonephritis, disease progression often involves more complex immune mechanisms, including prominent T cell infiltration or B cell activation. As a result, the model may underrepresent other relevant pathogenic pathways

## Data Availability

Not applicable.

## Abbreviations

ACE: Angiotensin-converting enzyme
BET: Bromodomain and extra-terminal domain
cGN: Crescentic glomerulonephritis
Crry: Complement receptor 1-related gene/protein y
DAF: Decay-accelerating factor
fms-I: C-fms kinase inhibitor
FSGS: Focal segmental glomerulosclerosis
GBM: Glomerular basement membrane
hKLK1: Human tissue kallikrein 1
IDO: Indoleamine 2,3-dioxygenase
IFN-γ: Interferon-γ
IKK2: IκB kinases β
ILC3s: Innate lymphoid cells
LN: Lupus nephritis
Mcl-1: Myeloid cell leukemia 1
MIDD: Monoclonal immunoglobulin deposition disease
MN: Membranous nephropathy
MSCs: Mesenchymal stem cells
NC1: Noncollagenous domain 1
NO: Nitric oxide
NTN: Nephrotoxic serum nephritis
NTS: Nephrotoxic serum
PEGs: Parietal epithelial cells
PGE2: Prostaglandin E2
PLA2R: Phospholipase A2 receptor
RA: Retinoic acid
RARα: Retinoic acid receptor-α
rhTM: Recombinant human thrombomodulin
SD: Sprague-Dawley
SLE: Systemic lupus erythematosus
STAT3: Signal transducers and activators of transcription 3
THSD7A: Thrombospondin type-1 domain-containing 7A
TNF: Tumor necrosis factor
Treg: Regulatory T
TRPC6: Canonical transient receptor potential-6
TWEAK: Tumor necrosis factor (TNF)-like weak
WKY: Wistar-Kytot
